# Particularities of a Cardiac Amorphous Left Ventricular Tumor in a Patient with Coronary Artery Disease—Diagnostic and Therapeutic Challenges: A Case Report and Literature Review

**DOI:** 10.3390/jcm13206092

**Published:** 2024-10-12

**Authors:** Caius Glad Streian, Cristina Tudoran, Raluca Elisabeta Staicu, Alina Gabriela Negru, Alexandra Laura Mederle, Claudia Borza, Ana Lascu

**Affiliations:** 1 Department VI Cardiology-Cardiovascular Surgery, “Victor Babeș” University of Medicine and Pharmacy of Timișoara, Eftimie Murgu Square No. 2, 300041 Timișoara, Romania; streian.caius@umft.ro (C.G.S.); alinanegru@umft.ro (A.G.N.); 2 Institute for Cardiovascular Diseases of Timisoara, “Victor Babeș” University of Medicine and Pharmacy of Timișoara, G. Adam Str. No. 13A, 300310 Timisoara, Romania; raluca.staicu@umft.ro (R.E.S.); lascu.ana@umft.ro (A.L.); 3 Advanced Research Center of the Institute for Cardiovascular Diseases, “Victor Babeș” University of Medicine and Pharmacy of Timișoara, Eftimie Murgu Square No. 2, 300041 Timișoara, Romania; 4 Department VII, Discipline of Cardiology, Internal Medicine II, “Victor Babeș” University of Medicine and Pharmacy of Timișoara, Eftimie Murgu Square No. 2, 300041 Timisoara, Romania; 5 Center of Molecular Research in Nephrology and Vascular Disease, “Victor Babeș” University of Medicine and Pharmacy of Timișoara, Eftimie Murgu Square No. 2, 300041 Timisoara, Romania; 6 Cardiology Clinic, County Emergency Hospital “Pius Brinzeu”, L. Rebreanu, No. 156, 300723 Timisoara, Romania; 7 Doctoral School Medicine-Pharmacy, “Victor Babeș” University of Medicine and Pharmacy of Timișoara, Eftimie Murgu Square No. 2, 300041 Timișoara, Romania; 8 Surgery Clinic, County Emergency Hospital “Pius Brinzeu”, L. Rebreanu, No. 156, 300723 Timisoara, Romania; alexandra.mederle@umft.ro; 9 Faculty of Medicine, “Victor Babeș” University of Medicine and Pharmacy of Timișoara, Eftimie Murgu Square No. 2, 300041 Timișoara, Romania; 10 Centre of Cognitive Research in Pathological Neuro-Psychiatry NEUROPSY-COG, “Victor Babeș” University of Medicine and Pharmacy of Timișoara, Eftimie Murgu Square No. 2, 300041 Timișoara, Romania; borza.claudia@umft.ro; 11 Department III Functional Sciences—Pathophysiology, “Victor Babeș” University of Medicine and Pharmacy of Timișoara, Eftimie Murgu Square No. 2, 300041 Timișoara, Romania; 12 Centre for Translational Research and Systems Medicine, “Victor Babeș” University of Medicine and Pharmacy of Timișoara, Eftimie Murgu Square No. 2, 300041 Timișoara, Romania

**Keywords:** amorphous cardiac tumor, echocardiography, trans-mitral excision, thrombophilia

## Abstract

**Background**: Cardiac calcified amorphous tumors (CATs) are rare non-neoplastic formations containing amorphous fibrinous material and calcifications. In our research, we present the case of a 42-year-old male patient who developed, during his 6-months monitoring for coronary artery disease, a left ventricular (LV) CAT raising diagnostic challenges. **Methods**: To gather additional information on CATs, we researched the international medical literature for scientific articles published with the full text in English, on PubMed, ResearchGate, Clarivate, and Google Scholar between 2020 and 2024. **Results**: Compared to most described cases, our patient was a young male, without mitral annular calcification or chronic renal disease, but he was suffering from chronic peripheral and coronary artery disease, and genetic testing revealed a higher risk for thromboembolic events. During 6 months, he developed a LV CAT of 4.5/3.5/3 cm. Although we found in the medical literature 16 case reports of patients with CAT, only six authors could specify a precise postoperative evolution of the CAT, most of them sustaining that if completely removed, it would not relapse, an aspect observed also in our patient during 3 years of follow-up. **Conclusions**: CATs are rare heart tumors with slow growth, but with a high embolization risk that raises diagnostic and therapeutic challenges.

## 1. Introduction

A cardiac calcified amorphous tumor (CAT) is seldom encountered in medical practice and was first mentioned by Reynolds et al. [1] in 1997. It refers to a non-neoplastic mass containing amorphous fibrinous material and calcified nodules [2,3]. This tumor is most frequently located in the mitral annulus, followed by the right atrium and ventricle, left ventricle and atrium, and the tricuspid annulus.

In most cases, it represents an incidental finding of a polypoid or mobile mass during an echocardiographic exam, raising challenges to distinguish it from a calcified myxoma or thrombi, cardiac osteosarcoma, or even from valvular vegetations or caseous calcifications of the mitral annulus [2,3].

Kerndt et al., in their meta-analysis, analyzed 73 patients with CATs and observed that on the echocardiography, 88% of the tumors were mobile, 91.8% represented singular masses, and all were described as hyperechoic structures [4]. An associated mitral annular calcification (MAC) was detected in 32.9% of the individuals. In 57.5% of all cases, CAT was attached to the leaflets and/or to the annulus leading to valvular dysfunction.

Usually, the first imaging modality that evidences the presence of masses in the heart chambers is transthoracic echocardiography (TTE). Although other imaging modalities such as chest-computed tomography (CCT), contrast-enhanced CCT, 18F-fluorodeoxyglucose (FDG)-positron emission tomography (PET), and magnetic resonance imaging (MRI) are beneficial for excluding a malignant tumor, these methods are not able to establish a clear CAT diagnosis [3]. A definite diagnosis of CAT is established based on the histopathologic examination of the resected intraventricular mass, which evidences amorphous material and calcifications [1]. Although most patients are asymptomatic, surgery is indicated due to the increased risk of embolization present in CATs, especially in the case of mobile lesions [2,3]. Some patients may claim symptoms such as palpitations, chest pain, syncope, dyspnea, and stroke, but often the first indication of a CAT is a systemic embolism [3,5].

Most studies indicate a favorable evolution after surgery and during follow-up, but in rare cases, mostly after the incomplete resection of the initial cardiac mass, recurrences have been reported [3,5].

Regarding CATs, a major problem is the necessity to establish the rate of their progress, and which factors could influence it. Since most of the CATs are detected after an embolic event in patients without a prior echocardiographic evaluation, the physicians are faced with large intracardiac masses, sometimes having several centimeters in diameter, often involving both myocardium and valvular leaflets, rendering the surgical intervention very challenging. In these conditions, it is frequently impossible to determine the moment when the tumor began to develop [6,7,8,9,10]. Even information regarding the frequency of recurrences and the period necessary for them to occur are missing [6].

Usually, a CAT is considered to grow slowly if it is not associated with other pathologies and risk factors [2]. Several studies pointed out that MAC represents a major risk factor for an accelerated progression of CATs, with some authors suggesting a period between 6 weeks and 1 year [2,11,12]. Other factors that could be associated with the development, and possibly the progression of CATs, are pre-existing valvular pathologies, end-stage renal disease (ESRD), and diabetes mellitus [12].

In this study, we aim to debate over the case of a young patient with multiple risk factors for atherosclerosis and coronary artery disease diagnosed with a relatively fast-developing CAT in the left ventricle, to follow the recurrence risk during a three-year follow-up after its surgical removal, and to highlight the factors that could influence the tumor′s evolution.

## 2. Case Presentation

We present the case of a 42-year-old male patient with a history of systemic hypertension and multiple risk factors for atherosclerosis such as smoking, hypercholesterolemia, and metabolic syndrome (altered basal glycemia, low HLD-cholesterol, overweight, and an increased abdominal circumference). He had a previous anteroseptal myocardial infarction in 2010, treated by thrombolysis, followed by an inferior myocardial infarction in 2017 for which he underwent a right coronary artery angioplasty followed by the implantation of three pharmacological active stents. Associated conditions were chronic peripheral artery disease, bilateral thromboangiitis obliterans, and chronic heart failure (CHF) with New York Heart Association (NYHA) class II. The patient was on stable treatment with atorvastatin 80 mg daily, cilostazol 100 mg twice daily, nebivolol 5 mg daily, and amlodipine 5 mg daily.

**Figure 1:**
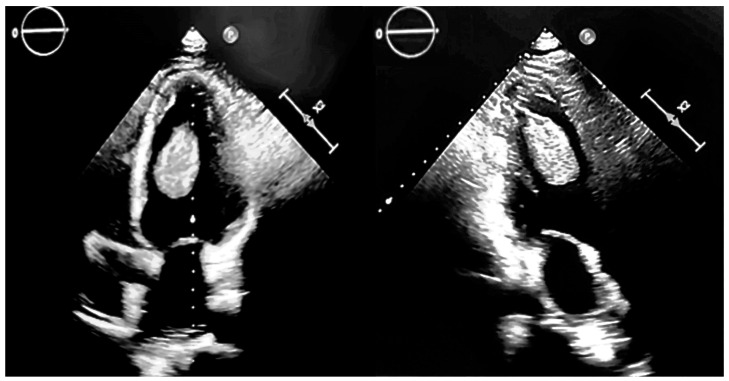
Echocardiographic appearance of a pediculated apical left ventricular tumor.

Considering his cardiac pathology, the patient was evaluated by his treating physician routinely at a 6-month interval. This evaluation also included a transthoracic echocardiographic (TTE) exam. In April 2021, a pediculated, echo-dense mass of approximately 4/3 cm in the left ventricle (LV) was detected by TTE and confirmed, subsequently, by transesophageal ultrasonography, Figure 1.

As thrombus was suspected initially, therapy with enoxaparin 0.6 mL twice daily was initiated for two weeks.

Although asymptomatic, due to the high mobility of the intracardiac mass, the risk of embolization was considered very high and the patient was referred to the Institute of Cardiovascular Disease in Timisoara for further investigations and the surgical removal of the tumor. Another reason for the surgical intervention was to establish a certain diagnosis, thus allowing appropriate management. At the clinical examination, he was overweight with a body mass index (BMI) of 27.68 Kg/m^2^, he had blood pressure values of 120/70 mmHg, and a systolic murmur in the mitral area. The electrocardiogram performed on admission, showed a sinus rhythm, intermediate QRS axis, ST segment depression of 1.5 mm in V2 and V3, and negative T waves in V1, DIII, and AVF. The TTE was performed at admission, detecting in the LV an echo-dense, pediculated mass with an inferior apical insertion, also confirmed by a transesophageal echography, Figure 1. Other findings were a dilated LV with an end-diastolic volume of 220 mL and an end-systolic volume of 125 mL, resulting in an ejection fraction of 38%, with lower septal akinesia, lower basal and median wall akinesia, mild functional mitral regurgitation, mild functional tricuspid regurgitation, and a right ventricle with a normal systolic function.

**Figure 2:**
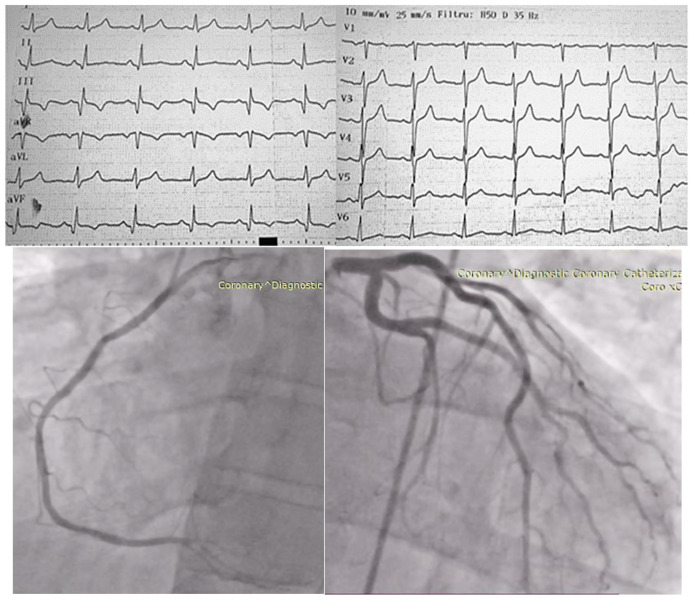
(**Above**)—Electrocardiogram indicating sinus rhythm, pathological Q wave in DIII, aVF, and negative T wave; (**Below**)—angiocoronarography of the right coronary artery (**left**), respectively, of the left coronary artery (**right**).

A coronary artery angiography was performed and it determined the patency of the three stents from the level of the right coronary artery and evidenced epicardial coronary arteries without significant angiographic lesions, see Figure 2.

The laboratory results at admission determined erythrocytes = 4.12 mil, leukocytes = 6.84 × 10^3^, platelets = 27,7000, hemoglobin = 13.0 g/dL, blood glucose = 107 mg/dL, creatinine = 1.11 mg/dL, uric acid = 4.8 mg/dL, creatine kinase = 72 U/L, creatine kinase MB = 15 U/L, prothrombin time = 17.3 s, INR = 0.97, and elevated liver transaminases (aspartate aminotransferase = 236 U/L and alanine aminotransferase = 426 U/L), which decreased during hospitalization.

**Figure 3:**
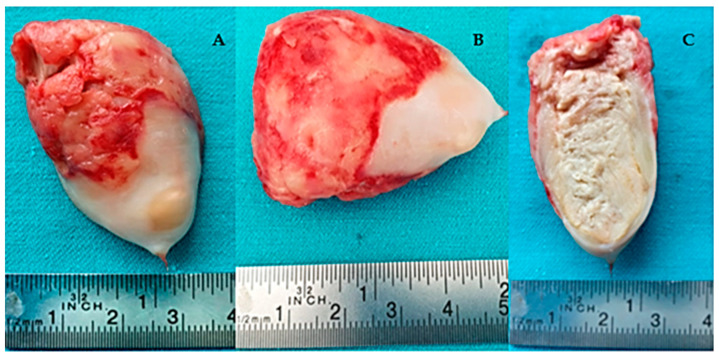
The macroscopic appearance of the tumor removed from the left ventricular: (**A**) frontal; (**B**) transversal; and (**C**) sagittal section through the tumor.

It was decided to perform surgery. The operation was conducted in normothermia using the St Thomas cardioplegic substance, administered anterogradely in two doses, with the cannulation of both the vena cava with a by-pass time of 42 min, ischemia time of 20 min, and a circulatory assistance time of 20 min. The excision of the tumor was performed through a trans-mitral approach. After left atriotomy, the tumor was visualized and it was extracted through digital mobilization from the outside of the LV. Macroscopically, it had the aspect of a thrombus, of an oval shape with a smooth surface and a short, small pedicle. A small area of thrombosis was observed on its surface. Its dimensions were 4.5/3.5/3 cm, see Figure 3. We also performed the left auricle′s ligature.

The patient required inotropic support with adrenaline and noradrenaline, but this medication could be withdrawn 24 h postoperatively. During the procedure, the patient presented an episode of atrial fibrillation, with a high ventricular frequency that was converted to a sinus rhythm with antiarrhythmic medication (Amiodarone).

**Figure 4:**
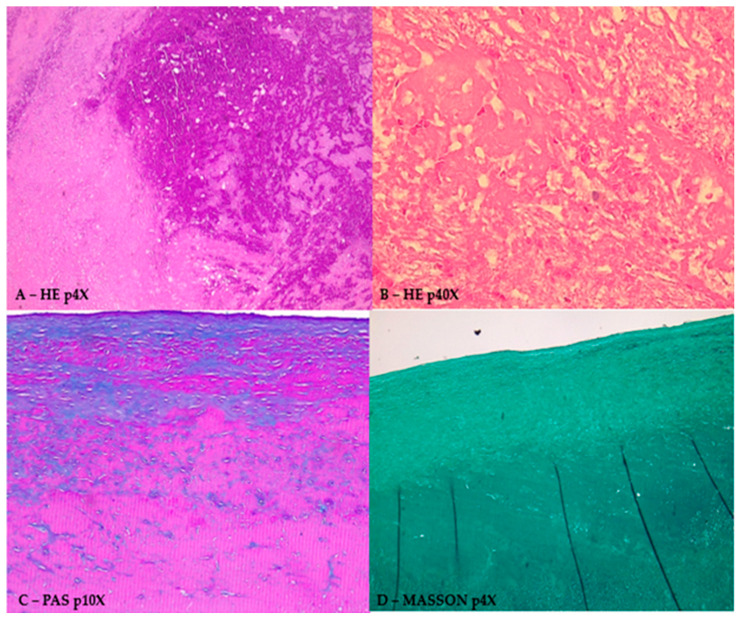
Histopathological exam of the excised tumor in various colorations. Legend: (**A**) HE coloration; p4X—eosinophilic amorphous substance with amorphous calcification; (**B**) HE coloration; p40X—eosinophilic amorphous substance with several red blood cells and amorphous calcification; (**C**) PAS alkaline blue coloration, p10X—blue zones indicating acid mucopolysaccharides; (**D**) Masson coloration p4X of the amorphous substance.

The histopathological exam of the tumor evidenced nodular conglomerations of an amorphous and fibrinous material with calcium deposits on an eosinophilic background, see Figure 4.

Postoperatively, the patient developed left basal pneumonia, treated with a combined antibiotic therapy (Cefort and Tazocin). The patient′s evolution was favorable and he was discharged at 5 days after surgery, receiving, besides his previous therapy, anticoagulant (Eliquis) and antiplatelet (Aspenter) drugs.

Subsequently, a genetic examination of the thrombophilia risk revealed a genetic profile favoring thromboembolic events and decreased fibrinolytic activity (MTFR A 1298C and PAI-1 4G/5G- heterozygotic forms), which was the reason why anticoagulant therapy was continued.

The patient was followed, on a 6-month basis, for 3 years, and no relapse of the tumor was observed.

## 3. Discussion

CATs are seldomly encountered in medical practice, as already debated in the first comprehensive review published on this topic by de Hemptine et al. [5], who were able to detect only 44 such tumors described in 20 years since the first mention of this type of tumor, and continuing with the newly published review of Takahashi et al. in 2024, who analyzed 106 cases until 2022. Considering the diagnostic pitfalls encountered during the management of our case, we researched the medical literature for case reports, published with the full text in English, on Clarivate, PubMed, ResearchGate, and Google Scholar in the last 4 years, aiming to identify other case presentations of CATs with similar characteristics and diagnostic challenges. As we followed-up our patient for three years, fearing a relapse of the CAT, we also researched tumor characteristics and evolution. We identified 16 case reports published between 2020 and 2024, as presented in Table 1. As already discussed in previous articles [5], CATs are more frequently encountered in middle-aged women, an aspect also observed in the cases analyzed in our research where there were 4 men (25%) and 12 women (75%) with a mean age of 68 years, in contrast to our patient who was of the male gender and 42 years old, diagnosed with coronary artery disease and thrombogenesis anomalies, but without MAC or CKD. Commonly (more than one third), CATs are located in the region of the mitral annulus, but in our case, it developed at the apex of the LV.

The pathogenesis of CATs is insufficiently specified and largely unknown. Several conditions such as valve diseases, particularly MAC, ESRD, diabetes mellitus, and coronary artery disease, were frequently reported to be associated with CATs [21]. Although CKD is considered one of the main factors influencing the development and the growth rate of CAT, from all the 16 articles described in Table 1, debating on patients with CATs, only three patients (two men and a woman), had CKD, and only one was on chronic hemodialysis [2,3,4]. Interestingly, in nine cases, the patient’s renal status was not mentioned. Considering that four of the described patients were over 80 years old, in these circumstances, we considered that the creatinine’s values were within the normal range and CKD was absent [5,6,7,8]. Various other etiologies favoring the occurrence of CATs were advanced, including abnormalities of the coagulation system, the development of an organized calcific mural thrombus, abnormalities of the calcium and phosphate metabolism, and chronic inflammation, as described in most of the analyzed papers [12,18,19]. One of these hypotheses on CAT pathogenesis is the formation of a thrombus due to the hypercoagulability, which is subsequently calcified as a consequence of an abnormal calcium and phosphorus metabolism, but in the analyzed papers, only Formelli et al. discussed this possibility as a consequence of polyglobulia [20]. In this context, our patient had a genetic abnormal thrombogenetic risk profile (MTFR A 1298C and PAI-1 4G/5G- heterozygotic forms), suggesting decreased fibrinolytic activity, thus resulting in an elevated risk for thrombo-embolic events.

CATs are frequently asymptomatic. As described by Takahashi et al. [22] in their recent comprehensive review, by analyzing individuals from all over the globe, most of the patients (75.5%) reported various symptoms, and less than a quarter were asymptomatic. More than half claimed dyspnea, around a quarter accused chest pain/chest discomfort, and less frequently, palpitations were encountered only in three subjects. Fewer claimed neurological symptoms (nine—syncope, eight—hemiparesis, three—transient ischemic attacks, dizziness or vertigo in two, and visual loss in six subjects), and some of them had uncharacteristic complaints such as general malaise—nine, a cough—seven, and fever in five patients. Similarly, in our review, the most frequent symptoms, encountered in about a quarter of patients, were dyspnea, chest pain, and acute stroke, followed by visual loss, palpitations, abdominal pain, syncope, and fatigue.

Multiple of these symptoms could be attributed to associated medical conditions or complications, especially to chronic heart failure or embolism. In the review of Takahashi et al., embolism occurred in about 30% (32 patients): half of them were cerebral infarctions, a quarter were pulmonary embolisms, and fewer had acute retinal artery occlusions (six subjects), acute myocardial infarction (two patients), and acute limb ischemia (two patients, one of them with both cerebral infarction and acute retinal artery occlusion) [22].

The most frequent location was the LV, in the region of the mitral annulus, with the CAT occurrence being favored by a previous MAC, and favored commonly by the calcium/phosphorus imbalances seen in ESRD or other metabolic disturbances [2].

In their review, Takahashi et al. highlighted that the size of CATs varies according to their location, from a smaller, mobile, linear, club-shaped, or spindle-type CAT, located in the mitral valve region, to a larger, expansive, immobile, tumor-like, or diffuse-type CAT in the right side of the heart. CATs in the left side of the heart are usually solitary and highly mobile. These authors suggested that end-stage renal disease could favor the development of multiple CATs, located on the mitral valve [22].

**Figure 5:**
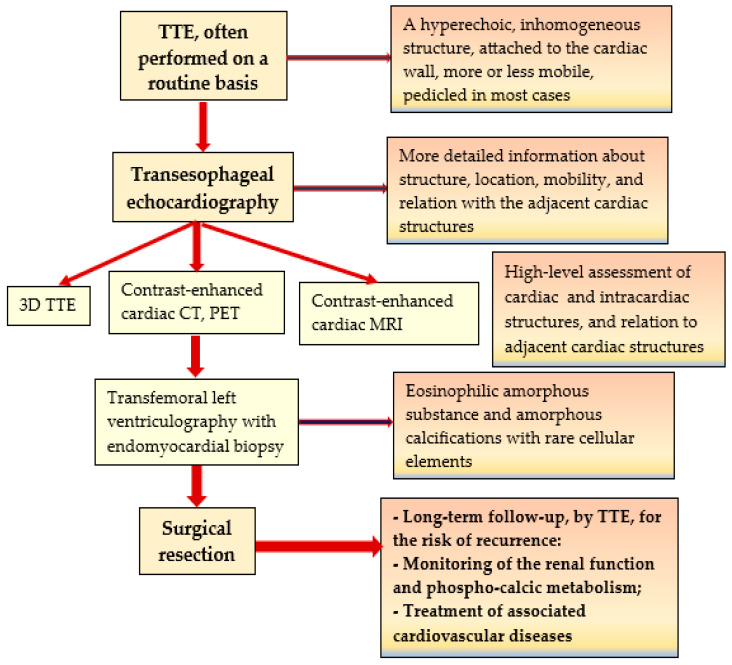
Algorithm for the diagnosis and management of intracardiac masses. Legend: CT—computed tomography; MRI—magnetic resonance imaging; PET—positron emission tomography; TTE—transthoracic echocardiography.

The presence of an intracardiac mass raises numerous diagnostic challenges. Most frequently, the possibility of a calcified thrombus is considered for the differential diagnosis of CATs. It is usually detected at a rutin TTE. A ventricular thrombus could be frequently seen in association with myocardial scars or severe LV dysfunction. The LV thrombus usually has a linear, smoother shape, commonly detected along the endocardial surface at the middle of the LV basis in the absence of an LV aneurysm. The transesophageal echocardiography allows a superior assessment of the intracardiac mass, and even more detailed information can be obtained by 3D echocardiography. On the MRI scans, the signal characterizing the thrombus appears different in concordance to the age of the thrombus. The contrast-enhanced MRI is helpful for the differential diagnosis between CATs and other cardiac tumors [1,2,23]. In our patient, it was particularly difficult to differentiate between a thrombus and a tumor, since the patient had previous myocardial infarctions. Following the challenges encountered during the diagnosis and therapy of this case, we developed an algorithm to simplify the management of patients with intracardiac masses, see Figure 5.

The growth rate of CATs is slow, but in the situation of an associated MAC, the growth rate of a CAT may accelerate. The larger the CAT is, the higher the risk that it could involve not only the LV wall, but also other structures, like the papillary muscles, the mitral valve, or one or two cusps of the aortic valve [2,14,16]. The involvement of the mitral or aortic valves could lead to valvular dysfunction, and to a higher intraoperative risk, sometimes even necessitating valve repair.

Unfortunately, the data on the prevalence, pathogeny, favoring factors, and evolution of CATs are very scant. Considering the high risk of embolization and the need for a histopathologic exam to establish a clear diagnosis, most authors have decided to perform surgery. However, to our knowledge, Ufuk et al. [3] is the only author who, after performing a transfemoral LV endomyocardial biopsy to establish a precise diagnosis of a CAT, decided to postpone surgery and followed-up the patient for 6 years, documenting a stable lesion. We did not find data regarding other treatment options, except surgery. In old, frail patients, with a reduced life expectancy and very high intraoperative risk, it would be an option to postpone surgery and to monitor the CAT on a regular basis, especially in slow-growing lesions with reduced mobility. The further development of imaging methods and of transvascular or minimally invasive procedures will allow, in the future, superior diagnostic possibilities and less invasive therapeutic options.

## 4. Conclusions

Although CATs are rare benign cardiac tumors, considering their high risk of systemic embolization, surgery is indicated. It is important to diagnose them timely and to monitor their growth rate and the factors that could accelerate it because a bigger tumor triggers a higher intra-operative risk due to the possible involvement of the mitral and/or aortic valves beside the LV wall.

## Figures and Tables

**Table 1:** Selection of case reports with CATs published between 2020 and 2024.

No.	Authors/Year	No. of Patients/Gender/Age	Associated Conditions	Location in LV	Symptoms	Renal Status	CAT Evolution
1.	Tsushima et al./2024 [7]	1/M/58	Hemodialysis	A mass attached to the posterior mitral leaflet and dense mitral annular calcification	Acute stroke	Heamodyalisis for 2 years	Unknown
2.	Hatori et al./2024 [13]	1/F/77	Systemic hypertension, lung cancer, coronary artery disease	A mobile mass in the LV outflow tract attached to the non-coronary and the right coronary aortic cusp	Dyspnea, chest pain	Creatinine = 0.85 mg/dL	Unknown
3.	Eizawa et al./2023 [8]	1/F/80	Calcified mitral annulus	Mobile mass originating from the mitral annulus of the posterior leaflet	Left eye vision loss due to an embolus at the bifurcation of the retinal artery	NR	Unknown
4.	Ufuc et al./2023 [3]	1/M/58	Not significant	Calcified, irregular tubular, and infiltrative mass in the inferior-basal segment of LV, extending in the mid-inferior wall, mid-inferior septum, and inferior papillary muscle	Chest pain, palpitations	NR	3 years
5.	Ghaballi et al./2023 [14]	1/M/37	Not significant	An irregular mass with a wide base attached to the atrial wall	Chest pain	NR	5 years
6.	Odujoko et al./2023 [15]	1/F/34	Type 1 diabetes mellitus, hypertension, Hashimoto thyroiditis, mesenteric ischemia, recent myocardial infarction of the posterolateral LV, moderate/severe coronary atherosclerosis, end-stage renal disease under hemodialysis	Masses on the mitral valve, the endocardium, and subendocardial portions of the left and right ventricles	Abdominal pain, gluteal pain, and diarrhea	Creatinine = 3.52 mg/dL, CKD	Autopsy finding
7.	Ushioda et al./2022 [9]	1/F/86	90% stenosis of the right coronary artery requiring coronary artery bypass, preserved ejection fraction, severe mitral annular calcifications	A mobile mass within the anterior annulus of the mitral valve	Episodes of syncope	NR	Unknown
8.	Endo et al./2022 [10]	1/F/71	Diabetes mellitus, multiple myeloma	A mobile, nonobstructive mass attached to the anterior leaflet of the mitral valve	Asymptomatic	NR	Unknown
9.	Kimura et al./2022 [16]	1/F/82	Mitral annular calcification, repeated strokes, dental infection	A calcified mass on the posterior leaflet	Acute stroke	NR	7 months
10.	Nishiguchi et al./2021 [12]	1/F/67	Stroke, systolic hypertension, hyperlipidemia	A pedunculated mass attached to the posterior leaflet of the mitral valve	Visual impairment	Creatinine = 1.45 mg/dL	5 months
11.	Kumar et al./2021 [2]	1/F/46	Chronic kidney disease stage 4, right thromboembolic frontoparietal infarct (2 months ago), coronary artery disease requiring coronary artery by-pass, heart failure with reduced ejection fraction	A pedunculated mobile mass attached to the intraventricular septum	Signs and symptoms of decompensated heart failure and decreased urine output	NR	Unknown
12.	Suzue et al./2021 [11]	1/F/83	Coronary artery bypass graft surgery, mitral annular calcification	Mobile, pediculated mass adherend to the posterior commissure of the mitral valve	Asymptomatic	Creatinine—0.43 mg/dL	4 months
13.	Koyama et al./2021 [17]	1/F/83	Aortic valve stenosis	Encapsulated mass between the noncoronary and the left coronary aortic cusp	Symptoms attributed to aortic valve stenosis	NR	Unknown
14.	Yamanaka et al./2020 [18]	1/F/82	Systemic hypertension, diabetes mellitus type 2, multiple myeloma, chemotherapy, MAC	An immobile, calcified mass on the mitral annulus	Asymptomatic	Creatinine = 0.61 mg/dL	18 months
15.	Okazaki et al./2020 [19]	1/M/67	Chronic hemodialysis, heart failure, systemic hypertension, gastric cancer, infectious endocarditis with Enterococcus faecalis	A mobile, cystic lesion with a mass inside the cyst attached to the apex of the LV wall	Fatigue, stomatitis, and diarrhea	ESRD	5 months
16	Formelli et al./2020 [20]	1/F/79	Systemic hypertension, polyglobulia	An echodense, not mobile left atrial mass	Ischemic stroke	NR	3 years

Legend: CKD—chronic kidney disease; ESRD—end-stage renal disease; LV—left ventricle; MAC—mitral annular calcification; NR—not reported.

## Data Availability

All data are mentioned in the manuscript.
